# Burden of Uncontrolled Hyperglycemia and Its Association with Patients Characteristics and Socioeconomic Status in Philadelphia, USA

**DOI:** 10.1089/heq.2020.0076

**Published:** 2020-12-30

**Authors:** Longjian Liu, Fengge Wang, Edward J. Gracely, Kari Moore, Steven Melly, Fengqing Zhang, Priscila Y. Sato, Howard J. Eisen

**Affiliations:** ^1^ Department of Epidemiology and Biostatistics, Drexel University Dornsife School of Public Health, Philadelphia, Pennsylvania, USA.; ^2^ Department of Family, Community & Preventive Medicine, Drexel University College of Medicine, Philadelphia, Pennsylvania, USA.; ^3^ Department of Psychology, Drexel University College of Arts and Sciences, Philadelphia, Pennsylvania, USA.; ^4^ Department of Pharmacology and Physiology, Drexel University College of Medicine, Philadelphia, Pennsylvania, USA.; ^5^ Division of Cardiology, Penn State University Heart and Vascular Institute, Hershey, Pennsylvania, USA.

**Keywords:** uncontrolled hyperglycemia, age effect, socioeconomic disparity

## Abstract

**Purpose:** To examine the burden of uncontrolled hyperglycemia in patients with diabetes mellitus (DM) and their characteristics in a large urban city.

**Methods:** A randomized sample of 4993 patients with DM ≥18 years old who received routine health care in a large university teaching hospital in the city of Philadelphia was analyzed. Uncontrolled hyperglycemia was classified as blood hemoglobin A1c >8%. The associations of uncontrolled hyperglycemia with sociodemographic and cardiovascular factors were analyzed using univariate and multivariate analysis methods.

**Results:** The results show that patients 18–54 years had the highest prevalence of uncontrolled hyperglycemia (36.0%), followed by those at age 55–64 (30.9%), 65–74 (22.9%), and ≥75 (20.6%) years (*p*<0.0001). Unadjusted hyperglycemia was significantly associated with patients with increased total cholesterol to high-density lipoprotein ratio (odds ratio [OR]=1.59, 95% confidence interval [CI]: 1.33–1.90, *p*<0.001), and with prevalent coronary heart disease (OR=1.39, 95% CI: 1.16–1.67, *p*=0.001). Patients living in neighborhoods with lower socioeconomic status (SES) had significantly higher uncontrolled hyperglycemia rates across the city (*r*=0.52, *R*^2^=0.27, *p*=0.03).

**Conclusions:** Findings of this study is one of the first studies to address that younger adults had higher rates of uncontrolled hyperglycemia. Further attention should be paid to the challenges of controlling DM in younger adults and patients who live in neighborhoods with lower SES.

## Introduction

Diabetes mellitus (DM) is a major public health concern that is approaching a new epidemic in both developing and developed societies. In the United States (U.S.), an estimated 30.2 million adults 18 years or older (12.2%) have DM. About 95% of people with DM are estimated to have type 2 DM (T2DM)^1^ that affects all parts of the body. DM can cause serious, potentially life-threatening complications, including cardiovascular complications.^2–6^ In the United States, of the largest 10 cities by population, the city of Philadelphia had the highest prevalence of DM (15.4%) in adults 18 years or older compared with the other nine largest cities (New York, Los Angeles, Chicago, Houston, Phoenix, San Antonio, San Diego, Dallas, and San Jose), all of which had DM rates below the U.S. average of 12.2%.^7^ In 2017, mortality from heart disease was 204 per 100,000 population in the city of Philadelphia, which was significantly higher than the average of 176 per 100,000 population in the state of Pennsylvania and the average of 165 per 100,000 population in the U.S. total population.^8^ Given the rapid increase in the number of populations with DM, intensive treatment for the disease becomes a critical step to reduce the burden of the disease and to prevent diabetic complications. To evaluate the disease treatment and prognosis, the American Diabetes Association (ADA) has developed its clinical practice recommendations. One of these is to actively monitor glycemic management using an appropriate measure, primarily plasma glycosylated hemoglobin A1c (HbA1c). The values of HbA1c indicate the recent status of glycemic control. An HbA1c level of 6.5% or higher on two separate occasions indicates a subject having DM. An HBA1c level of >7% to >8% has been used as an indicator of uncontrolled hyperglycemia in patients with DM.^9,10^ Most studies have reported the incidence and prevalence of DM, but less attention has been paid to evaluating the treatment and disease control status at both individual and community levels. In the study, we aimed to fill in these gaps by evaluating the burden of uncontrolled DM and examining its associations with socioeconomic status (SES) and cardiovascular risk factors in patients with DM in the city of Philadelphia.

## Methods

### Study design and population

We conducted a hospital-based cross-sectional study using a random sample of electronic health records (EHRs) data that were taken from one of the largest university teaching hospitals in the city of Philadelphia. The hospital's EHRs system was established in 2007. This technique of recording daily health care practice is qualified by the federal 2009 Health Information Technology for Economic and Clinical Health (HITECH) Act. To maximally apply the information system for research, we enrolled 4993 patients 18 years or older who were diagnosed as having diabetes and received health care between January 1, 2011 and December 30, 2015 in the hospital. Of the participants, 4911 (98.36%) had valid ICD-9 or ICD-10 recodes, including 2.54% diagnosed with type 1 DM (T1DM; *n*=127), 95.17% with T2DM (*n*=4752), and 0.64% with hybrid diabetes (a form of DM that has characteristics of both types 1 and 2 [*n* = 32]). The remaining 82 cases (1.64%) with missing ICD-9 or ICD-10 records were classified as T2DM according to their ages at the disease occurrence and medication treatment. For all the participants, their latest physical examination (weight, height, blood pressures, and major comorbidities) and clinical biomarker measures (i.e., plasma HbA1c, total cholesterol [TC], low-density lipoprotein [LDL] cholesterol, high-density lipoprotein [HDL] cholesterol and triglycerides [TG], and serum creatinine) were extracted from the EHRs. The study protocol and data collection were reviewed and approved by the Drexel University Institutional Review Board (no. 1607004678).

### Key study variables

#### Outcome

Uncontrolled hyperglycemia: Patients with uncontrolled HbA1c level were defined by the cutoff value of HbA1c >8% (IFCC: >64 mmol/mol). The selection of this cutoff point is based on the guidelines from the ADA, the American College of Physicians, the Association of Clinical Endocrinologists, the American College of Endocrinology, and several published works.^11–14^

#### Exposures and covariates

Participants' demographic variables (age, sex, and race/ethnicity) and their residential addresses (for the purpose of linking with census tract-level social determinants, see the section of Measure of SES) were collected. The latest measures (as the measure closest in time to the measures of HbA1c) of weight, height, systolic and diastolic blood pressures (SBP and DBP), serum lipids profile, creatinine, and medical conditions of hypertension (HTN), coronary heart disease (CHD), heart failure (HF), and stroke were collected from the EHRs.

#### Measure of SES

The EHRs system primarily records patients' clinical examination, diagnosis, laboratory tests and treatment status, whereas information on patients' SES at neighborhood levels is not collected. To integrate the EHRs dataset with patients' neighborhood SES, we used census tract-based SES data from 2010 to 2014 American Community Surveys (ACS). Variables from the ACS were selected to represent SES within the domains of overcrowding, housing, residential stability, educational attainment, employment, income, and wealth. A total of 16 variables was included to develop an SES summary score (Supplementary Table S1). Principal factor analysis with a varimax rotation was used to create the SES score based on the methods described elsewhere.^15,16^ For the SES summary score, we used factor 1 that explained the majority (48%) of the variation. The SES summary score was created as a weighted score by multiplying the standardized (z-score) for each variable by the factor weight and then sum all weighted variables. The estimated census tract-based SES score was further used to estimate mean SES scores across 18 neighborhoods (i.e., districts) across the city of Philadelphia.^17^ It should be noted that these geographic districts are created by the City Government to support better planning for the city of Philadelphia as a whole. Several health studies have applied this geographic classification, such as the city's Community Health Assessment that has been conducted annually since 2014. We apply this classification for adding comparable data analysis to the city's population health studies and to address a potential serious public health issue among diabetic patients with poorer HbA1c control status. In the analysis, neighborhoods with a higher SES score indicate a better SES, and a lower SES score for a worse SES.

### Statistical analysis

**FIG. 1.**
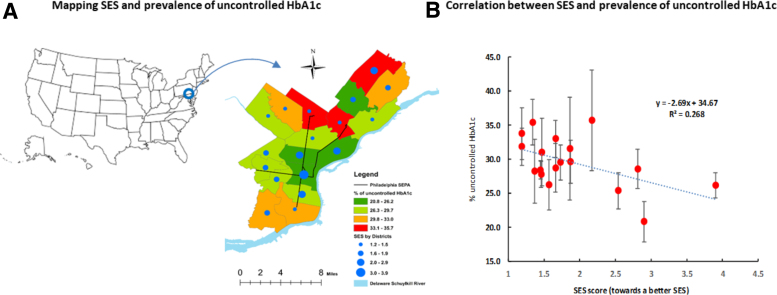
Mean SES score and prevalence of uncontrolled HbA1c. HbA1c, hemoglobin A1c; SES, socioeconomic status. **(A)** Mapping SES and uncontrolled HbA1c. **(B)** Correlation between SES and HbA1c >8%.

A serial analysis was conducted. In the first set of analyses, we described the overall characteristics of participants with controlled and those with uncontrolled HbA1c. Student's *t*-test for mean differences in continuous variables and chi-square test for rate differences in categorical variables were used. In the second set of analyses, we examined the associations between uncontrolled HbA1c and lipid profiles by age. In the analysis, dyslipidemias were categorized according to clinical guidelines in patients with diabetes for TC (≥200 mg/dL, i.e., SI units, TC ≥5.17 mmol/L), LDL (≥100 mg/dL, i.e., ≥2.59 mmol/L), HDL (<50/<40 mg/dL, or 1.29/1.03 mmol/L for women/men), and TG (≥150 mg/dL, i.e., ≥1.69 mmol/L). In the third set of analyses, we used logistic regression to estimate adjusted odds ratios (ORs) (95% confidence interval [CI]) of age, SES, dyslipidemia, and medical conditions for uncontrolled HbA1c. In model 1, we adjusted for age, sex, and race/ethnicity. In model 2, we adjusted the covariates used in model 1 plus body mass index (BMI in four groups: BMI <18.5, 18.5–24.9, 25–29.9, and ≥30 kg/m^2^) and medical history. Multiplicative interaction effects of age with SES, TC/HDL, LDL, HTN, CHD, stroke, and HF on the odds of uncontrolled HbA1c were tested as well. In the logistic regression analyses, *p*-values were estimated based on maximum likelihood ratio tests. In the fourth set of analyses, we depicted the distributions of uncontrolled HbA1c rates and its correlation with SES using an ecological analysis approach by testing the correlation between the average SES and uncontrolled HbA1c rates across the 18 neighborhoods (i.e., a neighborhood level analysis, *n*=18) (Fig. 1).

### Sensitivity analysis

Three sensitivity analyses were conducted. First, we repeated our analysis using the cutoff value of HbA1c >7% as the definition of uncontrolled HbA1c. Second, to avoid potential bias owing to the inclusion of different types of diabetes (type 1, type 2, or hybrid DM), we repeated our analysis by excluding T1DM (*n*=127), hybrid diabetes (*n*=32), and those with missing ICD-9 and ICD-10 codes (*n*=82) from the multivariate logistic regression analyses. Third, we repeated the correlation analysis between the average SES and uncontrolled HbA1c rates across the 18 neighborhoods by excluding T1DM, hybrid diabetes, and unspecified cases owing to missing ICD-9 or ICD-10 codes.

All data analyses were conducted using SAS version 9.4 (SAS Institute, Inc., Cary, NC). Differences in uncontrolled HbA1c across neighborhoods/districts are depicted using GIS (Geographic Information System, version 10.5). Statistical significance was defined as a *p*<0.05 in two-sided tests.

## Results

### Characteristics of participants

The mean (standard deviation) age of the participants was 60.8 (12.3) years in men and 61.7 (14.7) years in women. The prevalence of uncontrolled HbA1c was 28.9% in men and 28.7% in women (gender difference, *p*=0.85). Table 1 shows that in the uncontrolled HbA1c group, the mean age was significantly younger than for those with controlled HbA1c (58.4 vs. 62.6 years old, *p*<0.0001). Patients with uncontrolled HbA1c had significantly higher means of BMI, SBP, TC, LDL, and TG, and a significantly lower mean HDL than those with controlled HbA1c. Patients with lower SES had significantly higher uncontrolled HbA1c rates. It appears there were no significant differences in HbA1c control status by sex, race/ethnicity, and medical conditions of HTN, CHD, stroke, and HF (Table 1).

**Table 1.** Characteristics of Participants by Hemoglobin A1c-Control Status

	By HBA1c—control status						
	HBA1c ≤8%			HBA1c >8%			
	N	Mean or %	SD	N	Mean or %	SD	p
Continuous variable							
HBA1c, %	3556	6.6	0.7	1437	10.2	1.8	<0.0001
Age, years	3556	62.5	13.7	1437	58.4	13.6	<0.0001
BMI, kg/m^2^	3519	33.0	8.2	1417	33.6	7.8	0.030
SBP, mmHg	3549	132.1	18.3	1429	133.4	19.3	0.025
DBP, mmHg	3548	78.1	11.2	1427	78.6	11.5	0.157
TC, mg/dL	3216	175.6	42.0	1252	182.8	46.3	<0.0001
HDL, mg/dL	3239	52.5	16.3	1287	50.7	17.6	0.001
TC/HDL	3203	3.6	1.3	1249	3.9	1.5	<0.0001
LDL, mg/dL	3198	96.6	36.0	1261	99.9	39.4	0.008
TG, mg/dL	3225	133.4	77.7	1267	158.3	93.3	<0.0001
Categorical variable							
Sex							0.852
Female	2163	71.3		870	28.7		
Male	1393	71.1		567	28.9		
Race/ethnicity							0.146
White	621	73.9		219	26.1		
AA	2641	70.8		1090	29.2		
Others	294	69.7		128	30.3		
SES (toward a better SES)							0.015
Low	849	69.3		376	30.7		
Medium low	896	70.4		376	29.6		
Medium High	844	70.4		355	29.6		
High	915	74.7		309	25.3		
HTN							0.416
No	597	70.1		255	29.9		
Yes	2959	71.5		1182	28.5		
Coronary heart disease							0.463
No	2929	71.4		1171	28.6		
Yes	627	70.2		266	29.8		
Stroke							0.148
No	3071	70.9		1263	29.1		
Yes	485	73.6		174	26.4		
HF							0.564
No	3221	71.3		1294	28.7		
Yes	335	70.1		143	29.9		

AA, African Americans; BMI, body mass index; HBA1c, hemoglobin A1c; HDL/LDL, high/low-density lipoprotein cholesterol; HF, heart failure; HTN, hypertension; *N*, total sample; SBP/DBP, systolic/diastolic blood pressure; SD, standard deviation; SES, socioeconomic status score; TC, total cholesterol; TG, triglycerides.

### Associations between uncontrolled HbA1c and dyslipidemia

Table 2 shows that patients with elevated TC and TG and decreased HDL had significantly higher prevalence of uncontrolled HbA1c than those with normal TC, HDL, TC/HDL ratio, and TG in patients 18–64 years old. However, these significant associations were only observed in TC/HDL ratio (*p*=0.01) and TG (*p*=0.049) among the older adults (age ≥65 years).

**Table 2.** Prevalence (%) of Uncontrolled Hemoglobin A1c by Lipid Markers and Age

	Uncontrolled HbA1c (>8%)							
	Age 18–64				Age ≥65			
	Case	N	%	SEP	Case	N	%	SEP
TC, mg/dL								
Normal	563	1888	29.8	1.1	293	1387	21.1	1.1
High	298	775	38.5	1.7	98	418	23.4	2.1
*p*-value				<0.001				0.31
HDL, mg/dL								
Low	411	1126	36.5	1.4	144	587	24.5	1.8
Normal	475	1575	30.2	1.2	257	1238	20.8	1.2
*p*-value				0.001				0.07
TC/HDL ratio								
Normal	645	2084	29.9	1.0	321	1554	20.7	1.0
High	244	601	40.6	2.0	69	243	28.4	2.9
*p*-value				<0.001				0.01
LDL, mg/dL								
Normal	455	1437	31.7	1.2	258	1130	22.8	1.2
High	409	1214	33.7	1.4	139	678	20.5	1.6
*p*-value				0.27				0.25
TG, mg/dL								
Normal	468	1668	28.1	1.1	276	1331	20.7	1.1
High	401	1006	39.9	1.5	122	487	25.1	2.0
*p*-value				<0.001				0.049

LDL are classified: Normal LDL: <100, high ≥100 mg/dL.

Low HDL: <50/<40 for F/M; normal HDL: ≥50/≥40 mg/dL for F/M.

Normal TG: <150, high ≥150; normal TC: <200, high ≥200 mg/dL.

Normal TC/HDL ratio: <4.4/<5 for F/M; high TC/HDL ≥4.4/≥5 for F/M.

Case, uncontrolled case number; SEP, standard error of proportion.

### Multivariable analyses

Table 3 shows that after adjustment for covariates (Model 2), patients 18–54 and 55–64 years had significantly higher odds of uncontrolled HbA1c than patients ≥75 years older. The corresponding ORs (95% CI) were 2.09 (1.64–2.66, *p*<0.0001) and 1.73 (1.38–12.17, *p*<0.0001), Model 2. Patients with lower SES values had significantly higher odds of uncontrolled HbA1c compared with the highest SES group (Q4), the corresponding ORs were 1.30 (1.07–1.59, *p*=0.01), 1.32 (1.08–1.62, *p*=0.006), and 1.26 (1.02–0.54, *p*=0.029) for Q1 to Q3, respectively. Patients with elevated TC to HDL ratio (OR=1.59, 1.33–1.90, *p*<0.0001), and with CHD (OR=1.39, 1.16–1.67, *p*=0.001) had significantly higher odds of uncontrolled HbA1c. No significant interaction effects of age with the SES, TC/HDL ratio, LDL, or HTN, CHD, stroke, and HF were observed (*p*>0.05).

**Table 3.** Adjusted Odds Ratio (95% Confidence Interval) of Age, Socioeconomic Status and Other Factors Associated with Uncontrolled Hemoglobin A1c

	Model 1			Model 2		
	OR	95% CI	p	OR	95% CI	p
Age (Ref: ≥75)						
65–74	1.14	0.92–1.42	0.241	1.11	0.87–1.41	0.39
55–64	1.76	1.44–2.15	<0.0001	1.73	1.38–2.17	<0.0001
18–54	2.17	1.78–2.66	<0.0001	2.09	1.64–2.66	<0.0001
SES (Ref: Q4—high)						
Q3 (medium high)	1.20	0.99–1.44	0.058	1.26	1.02–1.54	0.029
Q2 (medium low)	1.21	1.01–1.45	0.039	1.32	1.08–1.62	0.006
Q1 (low)	1.23	1.03–1.48	0.024	1.30	1.07–1.59	0.010
Dyslipidemia						
TC/HDL (H vs. N)	1.56	1.33–1.84	<0.0001	1.59	1.33–1.90	<0.0001
LDL (H vs. N)	0.99	0.87–1.14	0.931	0.91	0.78–1.05	0.203
Medical condition						
HTN (yes vs. no)	1.26	1.05–1.50	0.011	1.13	0.93–1.38	0.22
CHD (yes vs. no)	1.32	1.12–1.56	0.001	1.39	1.16–1.67	0.001
Stroke (yes vs. no)	1.03	0.85–1.25	0.763	0.96	0.78–1.18	0.69
HF (yes vs. no)	1.27	1.03–1.57	0.027	1.10	0.87–1.40	0.41
Interaction effect^*^						
Age^*^SES	1.00	0.73–1.36	0.994	0.96	0.69–1.34	0.81
Age^*^TC/HDL	1.08	0.75–1.55	0.689	1.13	0.77–1.65	0.53
Age^*^LDLC	1.27	0.96–1.69	0.100	1.21	0.90–1.63	0.21
Age^*^HTN	1.43	0.94–2.17	0.096	1.42	0.88–2.30	0.15
Age^*^CHD	1.21	0.87–1.68	0.270	1.29	0.90–1.84	0.17
Age^*^stroke	0.99	0.67–1.44	0.944	1.08	0.72–1.63	0.71
Age^*^HF	0.72	0.47–1.09	0.120	0.70	0.44–1.11	0.12

Model 1: adjusted for age, sex, and race, except for OR by age groups in which sex and race were adjusted.

Model 2: adjusted for covariates M1 + BMI (four groups).

^*^ Interaction terms: age in two groups (<65 vs. ≥65) multiplied by each tested variable.

CHD, coronary heart disease; CI, confidence interval; LDL, low-density lipoprotein cholesterol; OR, odds ratio; TC/HDL, ratio of total cholesterol to high-density lipoprotein cholesterol.

### The association between SES and uncontrolled HbA1c across neighborhoods

Figure 1 provides the distributions of the prevalence of uncontrolled HbA1c and mean SES scores across the city's 18 neighborhoods (districts) (Fig. 1A) in Philadelphia, and an inverse correlation between increased SES scores and decreased uncontrolled HbA1c rates (correlation coefficient, *r*=0.52, *p*=0.03). An estimated 26.8% of the variations in the prevalence of uncontrolled HbA1c rates could be explained by the variations of the mean differences in SES (*R*^2^=0.268).

### Sensitivity analyses

Finally, we repeated our analysis using the cutoff value of HbA1c >7% (i.e., >53 mmol/mol) as the classification of uncontrolled HbA1c. As expected, the lower cutoff of HbA1c led to an increased number of individuals who were classified as having uncontrolled HbA1c. However, the associations of uncontrolled HbA1c with ages, dyslipidemia, and medical history of disease were consistent with those found when we used HbA1c >8% (i.e., >64 mmol/mol) as the cutoff value. In the second sensitivity analysis, we repeated our analysis by excluding those with T1DM (*n*=127), hybrid diabetes (*n*=32), and those with missing ICD-9 and ICD-10 cords (*n*=82). The results from the rest of sample size (*n*=4752, 95% of the total sample size) are also consistent with the results using data from the total participants (*n*=4993) at participant individual level analysis. Similarly, findings from a repeated neighborhood-level correlation analysis between the average SES and uncontrolled HbA1c rates among those with exclusion of T1DM, hybrid diabetes, and missing ICD-9 or ICD-10 codes are consistent with the findings from the total participants. Therefore, we present the results from the total sample size analysis.

## Discussion

Main findings of the study emphasize that (1) almost one third (29%) of the total participants with diabetes did not meet a clinical treatment goal of control HbA1c ≤8%. (2) Younger adults had significantly higher prevalence of uncontrolled HbA1c than older adults. Uncontrolled HbA1c disproportionately affected participants who lived in neighborhoods with lower SES. (3) Uncontrolled HbA1c was significantly associated with serum dyslipidemia (TC/HDL ratio) and the prevalent CHD.

HbA1c reflects average glycemia over ∼3 months. Furthermore, because the measurement of HbA1c does not request fasting blood sample, HbA1c concentration has been applied as a valid indicator to assess glycemic control status. Findings from a number of studies show that control elevated HbA1c plays a pivotal role in the disease management of DM and preventing patients from the development of cardiovascular complications. Different studies have applied different cutoff values of HbA1c to assess hyperglycemia control.^10,13,14,18–21^ In our study, based on the criteria recommended by the ADA, we applied HbA1c >8% to classify patients with uncontrolled hyperglycemia in patients with DM because the majority of the participants in the study sample were African Americans and had high comorbidities of HTN (83%), CHD (18%), stroke (13%), and HF (10%); in those if a more stringent HbA1c treatment goal, such as HBA1c <6.5% or <7%, is considered it would be inappropriate or difficult to achieve.^9,10,12^ Findings from the Action to Control Cardiovascular Risk in Diabetes (ACCORD) study showed that an intensive glycemic target (i.e., HbA1c <6.5%) may put patients at a high risk of hypoglycemia or a significant therapeutic burden.^21^

Age-related differences in hyperglycemia control have been observed in several studies.^22,23^ However, our result was one of the first studies that observed younger adults had worse hyperglycemia control than those 65 years and older.^24,25^ This raises an important question: why younger adults have poorer control of the disease than the older? Several possible factors may partly explain the age difference. For example, older adults may be highly motivated to take part in diabetes screening and accept physicians' recommendations for self-care practice and adherence to medications than younger adults. Potential different pathophysiology by ages may exist as well. For example, D'Adamo and Caprio suggest that young adults with T2DM may have a more severe form of the disease. They may have a higher degree of insulin resistance to current treatment modalities.^26^ Further studies are needed to examine the age differences. Given the increase in the prevalence of DM in the city and nationwide in the United States, the pattern of age differences in hyperglycemia control should be paid a serious attention.

Our study indicated that uncontrolled HbA1c levels were associated with increased odds of dyslipidemia and CHD. These findings are consistent with previous reports.^1,14,19^ The mechanism by which DM increases the risk of dyslipidemia and cardiovascular disease is relatively well studied. For example, insulin is the principal antilipolytic regulator to stimulate lipolysis. Without its action, lipolysis in adipose tissue will increase. Most patients with DM have deficient insulin production or impaired insulin action. One of the important contributions of this study is that the study extended the previous studies by addressing the significant associations of uncontrolled HbA1c with poorer SES at patients' individual levels, and across the neighborhoods at district levels of the city. Potential neighborhood disparities, such as access to health care service, healthy foods, and the effects of environmental injustice may play a critical role in the disparities of the disease control across the different neighborhoods.^27–29^

The study has several advantages. First, the study was designed by using data from the EHRs system. Beyond traditional clinical studies by collecting data from clinical chart reviews or surveys, data from the EHRs minimize information bias, increase the sample size, and are cost-effective. This new research approach greatly enhances the capacity of research and health evaluation using data from the real world.

Second, we integrated clinical data with socioeconomic contexts at neighborhood levels through data from existing community-based surveys. This integration extends the use of EHRs and allows us to address health problems and disparities at both individual and neighborhood levels. On the contrary, there are several limitations in the study. First, the study participants were recruited from a single university teaching hospital although the distributions of patients cover all the 18 neighborhoods of the city. Potential selection bias may have occurred. Second, findings of the study cannot be interpreted for any causal associations because of the nature of a cross-sectional study design. Third, several other biomarkers for the assessment of disease prognosis such as C-reactive protein, homocysteine, and so on, were not included in the analysis because these variables had a high proportion of the participants with missing values in their measures. Further studies are warranted.

Despite these limitations, as discussed previously, findings from the study highlights that almost one third of DM patients with hyperglycemia had a poorly controlled HbA1c level. Of them, younger adults had the highest uncontrolled HbA1c rate. Uncontrolled HbA1c were significantly associated with people living in neighborhoods with lower SES. Great efforts to control the disease are expected to play a role in eliminating the observed disparities in the disease management at patient individual and community levels. Although a cross-sectional study design does not support a causal relationship, these results suggest that such efforts could also provide risk reduction of cardiovascular disease in patients with diabetes.

## Supplementary Material

Supplemental data

## Abbreviations Used

AA: African Americans
ACS: American Community Surveys
ADA: American Diabetes Association
BMI: body mass index
CHD: coronary heart disease
CI: confidence interval
DBP: diastolic blood pressure
DM: diabetes mellitus
EHR: electronic health record
GIS: Geographic Information System
HbA1c: hemoglobin A1c
HDL: high-density lipoprotein
HF: heart failure
HITECH: Health Information Technology for Economic and Clinical Health
HTN: hypertension
OR: odds ratio
SBP: systolic blood pressure
SD: standard deviation
SEP: standard error of proportion
SES: socioeconomic status
TC: total cholesterol
TG: triglycerides
