# Supplementary material for: Burden of Uncontrolled Hyperglycemia and Its Association with Patients Characteristics and Socioeconomic Status in Philadelphia, USA
Source: Health Equity. 2020 Dec 30;4(1):525–32. doi: 10.1089/heq.2020.0076 (PMC8175259; doi:10.1089/heq.2020.0076)
Supplement: Supplemental data [file Supp_Table1.docx]

Supplementary Table 1: Sixteen variables used in creating socioeconomic status score using Principal components analysis and factor analysis

| **Domain** | **Variable** | **Description** |
| --- | --- | --- |
| HOUSING | X6 | Percent of occupied housing units |
| HOUSING | X7 | Percent of housing units that are owner occupied out of total housing units |
| HOUSING | X8 | Median value of occupied housing units |
| HOUSING | X9 | Percent of housing units without telephone |
| HOUSING | X10 | Percent of housing units without vehicle |
| RESIDENTIAL STABILITY | X11 | Percent living in same house in 1995 (Census 2000) or 1999 (ACS) |
| EDUCATION | X12 | Percent of person 25 or older with at least high school education |
| EDUCATION | X13 | Percent of persons 25 or older with at least a Bachelor’s degree |
| EMPLOYMENT | X14 | Percent unemployed among civilians 16 and over in the labor force |
| EMPLOYMENT | X15 | Percent of civilians 16 and over not in the labor force |
| OCCUPATION | X16 | Percent with management, professional, and related occupation |
| INCOME/ WEALTH | X17 | Median household income |
| INCOME/ WEALTH | X18 | Percent households with household income>$50,000 |
| INCOME/ WEALTH | X19 | Percent of households with interest, dividends, or net rental income |
| INCOME/ WEALTH | X20 | Percent of households with public assistance |
| INCOME/ WEALTH | X21 | Percent of persons below the poverty level |
